# Cancer Stem Cells: Emergent Nature of Tumor Emergency

**DOI:** 10.3389/fgene.2018.00544

**Published:** 2018-11-16

**Authors:** Yaroslav R. Efremov, Anastasia S. Proskurina, Ekaterina A. Potter, Evgenia V. Dolgova, Oksana V. Efremova, Oleg S. Taranov, Aleksandr A. Ostanin, Elena R. Chernykh, Nikolay A. Kolchanov, Sergey S. Bogachev

**Affiliations:** ^1^Institute of Cytology and Genetics, Siberian Branch of the Russian Academy of Sciences, Novosibirsk, Russia; ^2^Department of Natural Sciences, Novosibirsk State University, Novosibirsk, Russia; ^3^The State Research Center of Virology and Biotechnology Vector, Koltsovo, Russia; ^4^Research Institute of Fundamental and Clinical Immunology, Novosibirsk, Russia

**Keywords:** cancer stem cell, TAMRA+ cells, induction of pluripotency, hypoxia, oxidative stress, xenobiotics, carcinogenesis, genes-markers of stemness

## Abstract

A functional analysis of 167 genes overexpressed in Krebs-2 tumor initiating cells was performed. In the first part of the study, the genes were analyzed for their belonging to one or more of the three groups, which represent the three major phenotypic manifestation of malignancy of cancer cells, namely (1) proliferative self-sufficiency, (2) invasive growth and metastasis, and (3) multiple drug resistance. 96 genes out of 167 were identified as possible contributors to at least one of these fundamental properties. It was also found that substantial part of these genes are also known as genes responsible for formation and/or maintenance of the stemness of normal pluri-/multipotent stem cells. These results suggest that the malignancy is simply the ability to maintain the stem cell specific genes expression profile, and, as a consequence, the stemness itself regardless of the controlling effect of stem niches. In the second part of the study, three stress factors combined into the single concept of “generalized cellular stress,” which are assumed to activate the expression of these genes, were defined. In addition, possible mechanisms for such activation were identified. The data obtained suggest the existence of a mechanism for the *de novo* formation of a pluripotent/stem phenotype in the subpopulation of “committed” tumor cells.

## Introduction

### Hallmarks of cancer: version 2.0

Malignant neoplasms have been known to medicine for several thousand years, and it can assuredly be stated that all this time science has tried to find and formulate the fundamental properties that determine the development of tumors *in vivo*. The evolution of our ideas regarding the processes of the onset and development of tumors has overcome a very long and difficult way. As the first steps, the primitive macroscopic anatomical descriptions had been made (Yelloly, 1809). With the progress in methodology and instruments development, they gave way to the similar microscopic ones (Creighton, 1882) and even later–to the first attempts to determine the functional properties of tumor cells *in vitro* (Carrel and Ebeling, 1928). At the late steps, we came to an understanding (well, at least we tend to think so) of the fundamental physiological and molecular-genetic processes of tumor development, which, finally, made it possible to formulate the “Hallmarks of Cancer.”

There are two main points of view on the significant signs of malignancy of cancer and its underlying unit–cancer cells. In the first case, it is asserted that the hallmarks of cancer comprise six biological capabilities acquired during the multistep development of tumors. The hallmarks constitute an organizing principle for rationalizing the complexities of neoplastic disease. They include (1) Self-Sufficiency in Growth Signals, (2) Insensitivity to Antigrowth Signals, (3) Evading Apoptosis, (4) Limitless Replicative Potential, (5) Sustained Angiogenesis, and (6) Tissue Invasion and Metastasis (Hanahan and Weinberg, 2000, 2011).

In the second case, the authors offer an alternative set of key characteristics that determine the malignancy of a cancerous tumor and cancer cells that form it. This variant includes (1) selective growth and proliferative advantages, (2) altered stress response favoring overall survival, (3) vascularization, (4) invasion and metastasis, (5) metabolic rewiring, (6) an abetting microenvironment, and (7) immune modulation (Fouad and Aanei, 2017).

It is easy to note that these two lists both quite clearly overlap, have also quite a fundamental difference. Thus, for example, the authors of the second model do not include immortalization in the list of significant properties that define the behavior of the tumor. This property, in fact, represents a fundamental, extra-hierarchical qualitative event, which, on the one hand, is itself not a manifestation of malignancy, yet, on the other hand, is indispensable for its development.

Since the hallmarks of cancer and cancer cells malignancy, as they are denoted by the authors cited above, seem to be excessively detailed, we in our scrutiny narrowed them down to three more general categories that define the malignant potential at the phenotypic level. The first is the proliferative self-sufficiency as a set of characteristics that provide uncontrolled tumor growth. It comprises both independence from external mitogenic stimuli and immunity to stimuli that cause cell cycle arrest or apoptosis. The second one is invasiveness. It combines such properties as the ability to lyse the basal membrane, an increased capacity for migration, and the ability to adapt to the tissue environment, which is initially uncharacteristic for the tumor cell. And the last, third category is multiple drug resistance. This one is, in fact, a part of a broader detoxification mechanism essential for the survival of cells under aggressive tumor conditions. We also excluded from nomenclature both immortalization (for the reason described above) and sustained angiogenesis (due to ultimate dependence on the tumor context–this feature is essential for solid forms only).

### Cancer stem cell: the objectives and subjectives of the paradigm

Along with the definition of the cancer cells malignancy hallmarks and understanding of the mechanisms of tumor progression, data on the high heterogeneity of the tumor cellular mass were accumulated. These data turned out to contradict, to a certain extent, the theory of clonal origin of tumors.

The clonal nature of tumors has been known for a long time: it was first shown for human lymphomas (Fialkow et al., 1967, 1970; Steele, 1970) and subsequently confirmed for other types of tumors (Baylin et al., 1976; Nowell, 1976). At approximately the same time, it was found that tumors are quite heterogeneous and consist of cells that differ, and sometimes to a great extent, both in phenotype, and in physiological, proliferative and tumor-initiating attributes. For glioblastomas, for example, it was shown that tumors contain variable proportions of actively proliferating and nonproliferating tumor cells and that up to 70% of the cells in these tumors are resting (nonproliferating) (Hoshino and Wilson, 1975). However, one of the most convincing and demonstrative essays in terms of evidence of the tumor cells population heterogeneity is the work of Lavrovsky et al. In this essay, multiple clones from several spontaneously established murine sarcomas of CBA, C3H, and Balb/c genotypes were obtained and described. The phenotype of these clones was shown to vary from highly tumorigenic to the so-called pseudonormal. The tumorigenic clones were characterized by rapid multilayer growth and almost complete independence of the serum content, while the pseudonormal ones demonstrated sensitivity to growth factors as well as contact inhibition and the ability to differentiate into adipocytes after prolonged arrest in G0 (Lavrovsky et al., 1992).

Thus, in the early 90s of the last century, a firm paradigm for tumor growth emerged in molecular oncology. It was claimed that tumor growth is driven by a small subpopulation of actively dividing cells, while the rest of the cellular mass, which constitutes the bulk of the tumor, is a kind of ballast formed as a result of high genetic instability of tumor cells (Pathak, 1990).

The further accumulation of knowledge on tumors development has revealed that the cellular composition of tumors is essentially determined by certain internal rules, similar to those for normal organs. As a logical consequence, the previous paradigm has been evolutionary updated. In accordance to this updated paradigm, the tumor is considered an aberrant organ, developing from a subpopulation of poorly differentiated tumor cells with an infinite proliferative potential. For such a type of cells the new term “cancer stem cell” (by analogy with normal stem cells) was invented. And despite the term first being used in 1980 (Mattox and Von Hoff, 1980), the targeted investigation of this phenomenon started only in this millennium, when the term acquired its final meaning as a definition for poorly differentiated cells with indefinite potential for self-renewal that drive tumorigenesis (Reya et al., 2001).

However, in contrast to normal stem cells with their quite objective and clearly formalized criteria of stemness, the definition of stemness for cancer cells remains generally problematic and the search for such criteria is one of the high priority tasks in molecular oncology.

Recently, it was found that a certain subpopulation of Krebs-2 ascites carcinoma cells has the inherent ability to internalize fragments of extracellular double-stranded DNA (hereinafter–TAMRA+ cells). This subpopulation also demonstrates such a fundamental property of cancer stem cells (CSCs) as the ability to induce upon transplantation the development of a new tumor with histological and cellular characteristics similar to the original one. Elimination of these cells leads either to the loss of the grafting potential by the transplant, or to the cure of mice from developed Krebs-2 ascites (Dolgova et al., 2012, 2013, 2014, 2016; Potter et al., 2016b, 2018). Thus, the ability to internalize extracellular double-stranded DNA can be referred to as a valid marker (or, at least, as one of) of cancer cells stemness.

### Induction of stemness: alpha and omega of tumor development?

The paradigm of CSC and aberrant organogenesis had resolved the issue of tumors heterogeneity in the context of their clonal origin. But a new question had arisen.

The clonal nature of tumors implies that the entire mass of the tumor is the progeny of a single cell. The rapid and extensive growth of a tumor mass inevitably should lead to a situation, when a progenitor cell, i.e., CSC, remains in the very center of a tumor. It, in turn, should apparently cause either the complete cessation of tumor growth, or such a slowing down of it that is, in fact, almost equivalent to cessation. The tumor growth observed both in experiments and in medical practice is possible only in the presence of an essentially large number of CSCs, more or less evenly distributed throughout the tumor volume. As it is shown by our numerous experiments on mice and cultures of human cancer cells, as well as by the results of other researchers, the content of CSCs in tumor tissue varies from a few hundredths of a percent to several percents, and they are dispersedly scattered throughout a tumor mass or in ascitic fluid (Dolgova et al., 2014; Potter et al., 2016a,b). This means that under regular conditions of tumor tissue development, one CSC ensures the existence and biological activity of about 100–1,000 tumor cells. Assuming all the above, the question is how such a pattern of the CSCs distribution is being formed during the tumor quasi-organ development from a single progenitor.

It is generally accepted that the source of new CSCs, as in the case of normal pluri-/multipotent stem cells, is symmetrical division, as a manifestation of one of the fundamental properties of stemness. The newly formed CSC easily leaves not only its original site of localization in the tumor, but also the formed tumor tissue itself and, without losing its malignant properties, can migrate either to other parts of the growing tumor quasi-organ or to distant parts of the body. In other words, symmetrical division of the progenitor provides a constant supply of new CSCs, which migrate from the primary niche to the periphery of the tumor, creating new growth foci there, and the utmost case of such migrations is metastases.

This hypothesis, which explains the ability of CSCs to increase their population by symmetrical division followed by migration, is supported, in part, by the results we obtained earlier. Daily we estimated the numbers of TAMRA+ cells in Krebs-2 ascites from its onset and until the death of the animals (14 days). A characteristic oscillation in the number of TAMRA+ cells within 3 days accompanied by an increase in the volume of ascitic fluid and the total mass of cancer cells was observed. Along this time span, the number of TAMRA+ cells increased 3-fold and then returned to the baseline. The following model was proposed to explain this observation. The first act of symmetrical division produces two equal CSCs. One of these new CSCs enters the second division producing two daughter cells that both still possess the ability to internalize the TAMRA-labeled DNA probe. After the third division, the progeny of CSC lose their ability to internalize DNA and the percentage of TAMRA+ cells returns to initial value (Potter et al., 2016a,b).

Nevertheless, there are numerous data that suggest the existence of another mechanism for the formation and maintenance of the CSCs population.

Thus, in the study cited above, we found a discrepancy that did not fit into the theory explaining the increase in the number of CSCs as a result of their symmetrical division. It was found that for the majority of the mice analyzed, days of a “peak value” were observed, when the amount of CSCs significantly exceeded the regular threshold values typical for the observed oscillation of the CSCs counts (Potter et al., 2016a,b).

In the also mentioned above work of Lavrovsky et al, the efficacy of tumor formation upon transplantation of the progeny of the obtained clones into syngeneic mice has been evaluated. It was shown that tumors develop both in the case of highly tumorigenic clones, with the properties of CSCs, and in the case of pseudonormal cells, which displayed properties of committed cells. The difference between tumorigenic and pseudonormal clones was only in the incidence of tumor formation and in the time lapse required for this (Lavrovsky et al., 1992).

It is also known that many of immortalized cell lines displaying a “normal” phenotype of committed cells, such as various 3T3 lines, for example, produce tumors upon transplantation into syngeneic or immunodeficient animals (Greig et al., 1985; Melchiori et al., 1992). In other words, the data presented suggest that upon transplantation of “committed” cells of 3T3 type, i.e. possessing an infinite proliferative potential, but not an undifferentiated phenotype, *in vivo* CSCs can emerge *de novo*, giving rise to a tumor. Recent evidences support such a model of “dynamic stemness” for, at least, melanomas. Melanoma cells might temporally acquire tumor-initiating properties or switch from a status of tumor-initiating cells to a more differentiated one depending on the tumor context (Tuccitto et al., 2016).

A number of other studies demonstrating the feasibility of tumor cells to transit in both directions from cells of stem-like phenotype to differentiated ones and back again have also been compiled and reviewed (ElShamy and Duhé, 2013; Campos-Sánchez and Cobaleda, 2015).

Numerous observations of “dynamic stemness” allow to hypothesize the emergent nature of, at least, a part of the CSCs population. Accordingly, it is logical to presume that their emergence is associated with certain conditions in the micro- and humoral-environment, leading to the activation of the signaling pathways required for the induction of pluripotent/stem phenotype. Such a hypothesis implies the possibility of a reversible switching of the malignant identity of tumor cells and explains the distribution pattern of CSCs throughout the tumor volume, including its distal regions.

### Hallmarks of stemness: pointing the targets

Assuming all the above, it is CSCs that are obviously to be responsible for the implementation of the “tumorigenicity program” and thus have to evince the properties of malignancy to the highest extent, while the role of the remaining mass of tumor cells is still rather speculative.

Previously we have isolated the enriched population of TAMRA+ cells, which, as mentioned above, display all the principal properties of CSCs, and have identified 167 genes overexpressed in these cells relative to TAMRA− cells (see Additional Table 1) (Potter et al., 2017).

In accordance to the proposed model of malignancy that consists of proliferative self-sufficiency, invasiveness and multiple drug resistance, we analyzed all these 167 genes with regard to their possible roles in realization of these fundamental properties. The existing data mining revealed that the genes involved in the formation of TAMRA+ cells malignancy differed in their significance based on their contribution to the one or several attributes of malignancy simultaneously. It also turned out that besides their role as known CSCs markers, a significant part of genes from the list were also markers of stemness in normal pluri-/multipotent stem cells involved in maintaining their stem phenotype.

Upon identification of genes principal for formation and maintenance of the malignant/pluripotent properties of cancer cells, we have attempted to reveal the possible mechanisms of activation of these genes as well as to deduce the conditions essential for such an activation. Analysis of published data has revealed the plausible influence of stress factors on activation of both the identified genes and stem-like phenotype of tumor cells itself. The following analysis of ChIP-Seq data gave us a clue to possible mechanisms of activating effect of “generalized cellular stress.”

## The yin and yang of pluripotency

In the following parts of the article we describe a number of well known and generally accepted statements based on multiple experiments with a wide range of models including cellular *in vitro* models, experimental animals and clinically obtained tumor samples. To prove the majority of these statements we refer to review articles. In cases when the model represents an individual and unique one, we describe it in more details.

### Proliferative self-sufficiency

As already mentioned, we consider proliferative self-sufficiency as a complex property. On the one hand, it is defined as the ability of a cell to maintain proliferation under conditions of inaccessibility or deficiency in external mitogenic stimuli. On the other hand, it reflects the ability to keep viability and avoid apoptosis despite the presence of pro-apoptotic signals. It can be achieved by a rather large set of mechanisms, from autocrine synthesis and secretion of growth factors and components of the extracellular matrix (reviewed in Hoelzinger et al., 2007) to blocking the internal mechanisms of the apoptotic program (reviewed in Mallard and Tiralongo, 2017). The main problem we encountered in the analysis and selection of genes contributing to this property is the dependence of the functional properties of their protein products on the overall gene-protein context in each particular case. Often the same protein can act both as a tumor suppressor and as a tumor inducer depending on the type of cells or conditions. As an example, we can refer to the gene *Perp*, which was overexpressed in TAMRA+ cells, and which we, nevertheless, could not relate to any of the groups due to the lack of direct evidence of its functional effect on the formulated properties. It was shown that in the case of invasive squamous cell carcinoma, *Perp* functions as a tumor suppressor (Kong et al., 2013), while the *Perp*^−/−^ mice were more resistant to papilloma development than those of the wild-type, that suggests its pro-oncogenic function (Marques et al., 2005). Moreover, it is a possible case when the protein product of a gene normally functions as a tumor suppressor, but upon the mutation its properties as a tumor suppressor are either lost or even inverted and it acquires pro-oncogenic function as it is shown, for example, for “gain-of-function” mutations of the p53 tumor suppressor gene (Vogiatzi et al., 2016). Since we did not have the opportunity to resolve all these of issues, we decided that the gene is to be included in a certain functional group if in principle there is evidence of its positive impact on the implementation of the corresponding property. As a result, we have selected 82 genes that one way or another participate in formation of the proliferative self-sufficiency of tumor cells (Table 1).

**Table 1:** Genes showing elevated expression in TAMRA+ Krebs-2 carcinoma cells relative to TAMRA− cells, the activation of which results in excessive proliferative activity or resistance to apoptosis.

	**Gene**	**Synonyms**	**Proving reference**
1	*Abca1*	ABC1, HDLDT1, TGD	Buechler et al., 2002
2	*Acpp*	ACP-3, ACP3, PAP	Liu et al., 2014
3	*Adrb3*	adrenergic beta-3-receptor	Granneman et al., 2005
4	*Aldh1a1*	ALDH1, PUMB1, RALDH1	Meng et al., 2014
5	*Alox15*	15-LOX-1	Deliri et al., 2011
6	*Amy1*	AMY1A	Mizuno et al., 2015
7	*Ankrd22*	MGC22805	Yin et al., 2017
8	*Arg2*	Arginase, type II	Sousa et al., 2010
9	*Atp6v0d2*	ATP6D2, FLJ38708, VMA6	Morimura et al., 2008
10	*Blnk*	BASH, bca, BLNK-s, Ly57, SLP-65, SLP65	Tan et al., 2001
11	*Bmper*	CRIM3, Cv2	Heinke et al., 2012
12	*Cacna1d*	CACNL1A2, CCHL1A2, CACH3, CACN4, Cav1.3	Chen et al., 2014c
13	*Ccr3*	CMKBR3, CC-CKR-3, CD193, CKR3	Miyagaki et al., 2011
14	*Cd5l*	API6, Spalpha	You et al., 2015
15	*Cd55*	DAF, CR, CROM, TC	Yin et al., 2015
16	*Cd200*	MOX1, MOX2, MRC, OX-2	Jung et al., 2015b
17	*Chrm1*	Acetylcholine receptor, muscarinic 1	Mannan Baig et al., 2017
18	*Clec11a*	SCGF, CLECSF3, LSLCL, P47	Hiraoka, 2008
19	*Cldn1*	ILVASC, SEMP1	Pope et al., 2014
20	*Col3a1*	EDS4A	Su et al., 2014
21	*Col6a2*	Collagen type VI alpha 2	Cheng et al., 2011
22	*Comp*	EDM1, EPD1, PSACH, MED, THBS5, TSP5	Hashimoto et al., 2003
23	*Cp*	Ceruloplasmin, ferroxidase	Alcaín and Löw, 1997
24	*Crabp2*	CRABP-II	Liu et al., 2016b
25	*Cyp7a1*	Cholesterol 7 alpha-monooxygenase	Liu et al., 2016a
26	*Cyp26a1*	CP26, CYP26, P450RAI, P450RAI1	Osanai et al., 2010
27	*Ddx3y*	DBY	Kotov et al., 2017
28	*Dusp23*	DUSP25, FLJ20442	Tang et al., 2010
29	*Eef1a2*	STN, STNL, EEF1AL, HS1	Sun et al., 2014
30	*Eif2s3y*	EIF2S3, EIF2G, EIF2, EIF2gamma	Li et al., 2016c
31	*Fam107a*	DRR1, TU3A	Asano et al., 2010
32	*Fblim1*	CAL, FBLP-1, migfilin	Zhao et al., 2009
33	*Fgfr1*	BFGFR, CD331, CEK, FLG, H2, H3, H4, H5, N-SAM, FLT2, KAL2	Katoh and Nakagama, 2014
34	*Fmnl2*	FHOD2, formin-like 2	Li et al., 2016a
35	*Gas6*	AXLLG, AXSF	Jaluria et al., 2008
36	*Gata6*	GATA-binding protein 6	Lin et al., 2012
37	*Gdf6*	BMP13, KFS, KFS1, SGM1	Pant et al., 2013
38	*Gpha2*	GPA2, ZSIG51	Huang et al., 2016
39	*Grb10*	Growth factor receptor-bound protein 10	Kazi and Rönnstrand, 2013
40	*Hpn*	TMPRSS1	Xing et al., 2011
41	*Igf1*	IGF-I, IGF1A, IGFI, somatomedin C	Kasprzak et al., 2017
42	*Igf2*	IGF-II, preptin, somatomedin A	Bergman et al., 2013
43	*Il10*	CSIF, IL-10, IL10A, TGIF	Masood et al., 1995
44	*Il17rb*	IL17BR, CRL4, EVI27, IL17RH1	Alinejad et al., 2016
45	*Itga9*	ALPHA-RLC, ITGA4L, RLC	Zhang et al., 2016a
46	*Itln1*	hIntL, HL-1, ITLN, LFR, omentin	Zhao et al., 2015
47	*Kcnq2*	BFNC, ENB1, HNSPC, KCNA11, Kv7.2, EBN, EBN1	Salyer et al., 2013
48	*Lass4*	CERS4, Trh1, LAG1 homolog, ceramide synthase 4	Chen et al., 2017
49	*Lhx4*	Gsh4	Cha et al., 2014
50	*Ltbp1*	TGF-beta1-BP-1	Tritschler et al., 2009
51	*Lyve1*	XLKD1, LYVE-1	Huang et al., 2003
52	*Maged2*	11B6, BCG1, HCA10, JCL-1, MAGE-D2, MAGED	Papageorgio et al., 2007
53	*Mmp2*	CLG4, CLG4A, TBE-1	Chen et al., 2016a
54	*Nfatc2*	NF-ATP, NFAT1, NFATp	Horsley and Pavlath, 2002
55	*Nrcam*	Bravo, NgCAM-related cell adhesion molecule	Conacci-Sorrell et al., 2005
56	*Nt5e*	CALJA, CD73, eN, eNT	Gao et al., 2014
57	*Nts*	Neuromedin N, pro-neurotensin/neuromedin	Hu et al., 2015
58	*Pde4d*	DPDE3	Powers et al., 2015
59	*Pdk4*	–	Leclerc et al., 2017
60	*Per2*	–	Wang et al., 2016c
61	*Pf4*	CXCL4, SCYB4	Kasper et al., 2007
62	*Pon1*	Arylesterase 1, ESA	Aldonza et al., 2017
63	*Prg4*	CACP, bG174L6.2, HAPO, JCAP, MSF, SZP	Oikawa et al., 2017
64	*Prok2*	BV8, KAL4, MIT1, PK2	Xin et al., 2013
65	*Pvrl1*	ED4, HVEC, CD111, CLPED1, HIgR, nectin, OFC7, PRR, PRR1, PVRR1, SK-12	Bojesen et al., 2012
66	*Rab15*	–	Matsuo et al., 2014
67	*Rab37*	–	Dobashi et al., 2009
68	*Rasgrp3*	CalDAG-GEFIII, GRP3	Nagy et al., 2014
69	*Rragd*	bA11D8.2.1	Sasaki et al., 2012
70	*S100a14*	BCMP84, S100A15	Wang et al., 2015
71	*Serpinb1a*	ELANH2, anti-elastase, EI, PI2	Seaborn et al., 2014
72	*Serpinb2*	PAI2, PLANH2, HsT1201	Tonnetti et al., 2008
73	*Slc2a4*	GLUT4	Garrido et al., 2015
74	*Slco4a1*	SLC21A12, OATP-E, OATP4A1	Ban et al., 2017
75	*Tal1*	TCL5, bHLHa17, SCL	Lacombe et al., 2013
76	*Tcf7l2*	TCF4, TCF-4	Shitashige et al., 2008
77	*Tdo2*	TDO, TPH2	D'Amato et al., 2015
78	*Thpo*	MGDF, MPLLG, TPO	Chou et al., 2012
79	*Tnfrsf13c*	BAFFR, CD268	Fu et al., 2009
80	*Tnn*	TNW, TN-N, TN-W	Chiovaro et al., 2015
81	*Trpv4*	CMT2C, OTRPC4, TRP12, VR-OAC, VRL-2, VROAC	Zhan et al., 2015
82	*Wnt5a*	WNT-5A	Zhou et al., 2017

*Genes symbols and synonyms are given in accordance to HGNC nomenclature*.

### Invasiveness and metastasis

Another fundamental property of malignant tumors is their ability of invasive growth and metastasis. This process commonly starts with proteolytic degradation of the basal membrane by metalloproteinases of various types, the increased expression of which is one of the main indicators of invasive tumor growth (reviewed in Jiang et al., 2015). Further, the metastasizing cell must have a number of specific properties. First, it should be able to exist in an unattached state while in the bloodstream or lymphatic vessel. This functional feature overlaps to a significant extent with the previous property to block the apoptosis, in this case–apoptosis caused by the detachment from matrix, the so-called anoykis (reviewed in Taddei et al., 2012). Second, metastasizing cell should be able to settle down and normally proliferate in the initially alien tissue environment, which can be attained through the increased expression of numerous molecules of cell adhesion, often specific for lymphoid cells (reviewed in Chong et al., 2012). And third, the cell should be able to avoid a tissue-specific immune response. This is usually being achieved, either, similarly to the previous case, by expressing specific surface markers, or by synthesizing and secreting immunosuppressive mediators and cytokines (reviewed in Kuol et al., 2017). Another important role in the invasion and metastasis is assigned to proteins that stimulate the migratory function of cells (reviewed in Bordeleau et al., 2014). This group was constituted of 64 genes promoting one or more of mentioned functional properties (Table 2).

**Table 2:** Genes showing elevated expression in TAMRA+ Krebs-2 carcinoma cells relative to TAMRA− cells, the activation of which results in invasive growth and metastasis.

	**Gene**	**Synonyms**	**Proving Reference**
1	*Abca1*	ABC1, HDLDT1, TGD	Zhao et al., 2016
2	*Abca13*	–	Araújo et al., 2016
3	*Acpp*	ACP-3, ACP3, PAP	Kirschenbaum et al., 2016
4	*Adamts2*	ADAM-TS2, ADAMTS-3, hPCPNI, NPI, PCINP	Akyol et al., 2015
5	*Aldh1a1*	ALDH1, PUMB1, RALDH1	Wang et al., 2017
6	*Alox15*	15-LOX-1	Kerjaschki et al., 2011
7	*Arg2*	Arginase, type II	Costa et al., 2016
8	*Asb4*	ASB-4, ankyrin repeat and SOCS box-containing 4	Au et al., 2014
9	*Bmper*	CRIM3, Cv2	Heinke et al., 2012
10	*Cacna1d*	CACH3, CACN4, Cav1.3, CACNL1A2, CCHL1A2	Alinezhad et al., 2016
11	*Ccr3*	CC-CKR-3, CD193, CKR3, CMKBR3	Jung et al., 2010
12	*Cd55*	DAF, CR, CROM, TC	Mikesch et al., 2006
13	*Cd200*	MOX1, MOX2, MRC, OX-2	Gorczynski et al., 2011
14	*Cldn1*	ILVASC, SEMP1	Mahati et al., 2017b
15	*Col3a1*	EDS4A	Su et al., 2014
16	*Col6a2*	Collagen type VI alpha 2	Cheon et al., 2014
17	*Comp*	EDM1, EPD1, PSACH, MED, THBS5, TSP5	Englund et al., 2016
18	*Cp*	Ceruloplasmin, ferroxidase	Kluger et al., 2004
19	*Cyp26a1*	CP26, CYP26, P450RAI, P450RAI1	Osanai and Lee, 2015
20	*Dock10*	ZIZ3, zizimin3	Westcott et al., 2015
21	*Dusp23*	DUSP25	Tang et al., 2010
22	*Eef1a2*	STN, STNL, EEF1AL, HS1	Xu et al., 2013
23	*Fam107a*	DRR1, TU3A	Le et al., 2010
24	*Fblim1*	CAL, FBLP-1, migfilin	Gkretsi and Bogdanos, 2015
25	*Fgfr1*	BFGFR, CD331, CEK, FLG, H2, H3, H4, H5, N-SAM, FLT2, KAL2	Jiao et al., 2015b
26	*Fmnl2*	FHOD2	Zhu et al., 2011
27	*Gas6*	AXLLG, AXSF	Wang et al., 2016a
28	*Gata6*	–	Belaguli et al., 2010
29	*Grb10*	Growth factor receptor-bound protein 10	Khan et al., 2015
30	*Gstm3*	GST5	Meding et al., 2012
31	*Hpn*	TMPRSS1	Tang et al., 2014
32	*Igf1*	IGF-I, IGF1A, IGFI, somatomedin C	Lei and Ling, 2015
33	*Igf2*	IGF-II, preptin, somatomedin A	Lira et al., 2016
34	*Il10*	CSIF, IL-10, IL10A, TGIF	Zeng et al., 2010
35	*Il17rb*	CRL4, EVI27, IL17RH1, IL17BR	Wu et al., 2015
36	*Itga9*	ALPHA-RLC, ITGA4L, RLC	Zhang et al., 2016a
37	*Ltbp1*	TGF-beta1-BP-1	Mercado-Pimentel and Runyan, 2007
38	*Lyve1*	XLKD1, LYVE-1	Prevo et al., 2001
39	*Maged2*	11B6, BCG1, HCA10, JCL-1, MAGE-D2, MAGED	Kanda et al., 2016
40	*Mmp2*	CLG4, CLG4A, TBE-1	Xuan et al., 2015
41	*Mycbpap*	AMAP-1	Sabe et al., 2009
42	*Myo1b*	myr1	Ohmura et al., 2015
43	*Nfatc2*	NF-ATP, NFAT1, NFATp	Jauliac et al., 2002
44	*Nrcam*	Bravo, NgCAM-related cell adhesion molecule	Zhang et al., 2017
45	*Nt5e*	CALJA, CD73, eN, eNT	Wang et al., 2008
46	*Nts*	neuromedin N, pro-neurotensin/neuromedin	Ye et al., 2014
47	*Pde4d*	DPDE3	Delyon et al., 2017
48	*Pdk4*	–	Leclerc et al., 2017
49	*Per2*	–	Li et al., 2016d
50	*Pon1*	Arylesterase 1, ESA	Aldonza et al., 2017
51	*Ppap2b*	PLPP3, LPP3, PAP-2b	Westcott et al., 2015
52	*Rasgrp3*	CalDAG-GEFIII, GRP3	Zeng et al., 2014
53	*S100a14*	BCMP84, S100A15	Wang et al., 2015
54	*Selp*	GRMP, CD62P, GMP140, PADGEM, PSEL	Stübke et al., 2012
55	*Serpinb2*	PAI2, PLANH2, HsT1201	Jin et al., 2017a
56	*Slco4a1*	SLC21A12, OATP-E, OATP4A1	Ban et al., 2017
57	*Tal1*	TCL5, bHLHa17, SCL	Correia et al., 2016
58	*Tcf7l2*	TCF4, TCF-4	Ravindranath et al., 2008
59	*Tdo2*	TDO, TPH2	D'Amato et al., 2015
60	*Tnn*	TNW, TN-N, TN-W	Chiovaro et al., 2015
61	*Tnxb*	TNXB1, TNXB2, TNXBS, XB, XBS	Hu et al., 2009
62	*Trpv4*	CMT2C, OTRPC4, TRP12, VR-OAC, VRL-2, VROAC	Lee et al., 2017b
63	*Vsig4*	Z39IG	Zhang et al., 2016c
64	*Wnt5a*	WNT-5A	Shojima et al., 2015

*Genes symbols and synonyms are given in accordance to HGNC nomenclature*.

### Drug resistance

One of important, if not the most important, problems in clinical oncology is the resistance of tumors to antitumor drugs. When in the 50s of the last century this phenomenon had started to be actively investigated, the drug resistance was believed to be an adaptive response that develops as a result of tumor cells selection under long-term exposure to a certain drug. It was generally accepted to associate the drug resistance with an elevated level of expression of enzymes responsible for xenobiotics metabolism, such as P450 family oxygenases (reviewed in Harvey and Morgan, 2014), and specific transmembrane transport proteins providing efflux of xenobiotics and their metabolites (reviewed in Chen et al., 2016b). However, more recent observations have revealed that very often drug resistance is initially intrinsic to a certain subpopulation of tumor cells and is associated not only with the above-mentioned causes (reviewed in Gottesman, 2002). The main effect of antitumor drugs is known to be associated with their either cytostatic or cytotoxic properties, which in turn are mainly realized through DNA damage and should activate apoptotic processes. Accordingly, activation of mechanisms allowing to overcome G1/S arrest or blocking the realization of the apoptotic program, increases the resistance of tumor cells to chemotherapy (Volm, 1998). Moreover, the DNA-damaging effect of chemotherapeutic agents is neutralized by the cellular systems of antioxidative defense (reviewed in Victorino et al., 2014). And, finally, in the very end of the last century, another mechanism of tumors drug resistance—the so-called *Cell-Adhesion Mediated Drug Resistance* (CAM-DR) was discovered (reviewed in Dalton, 1999). This mechanism, in fact, represents a complex adaptive response that comprises the increased resistance to apoptosis due to anti-apoptotic signals from integrins (Damiano, 2002), reduced tumor permeability for chemotherapeutic agents (Kerbel et al., 1996; Grantab and Tannock, 2012), and formation of syncytium, which also leads to increased drug resistance (Lu and Kang, 2009; Nagler et al., 2011; Mittal et al., 2017). We found the evidences of anticancer therapy resistance inducing activity for 38 genes (Table 3).

**Table 3:** Genes showing elevated expression in TAMRA+ Krebs-2 carcinoma cells relative to TAMRA− cells, the activation of which results in increased resistance of cells to xenobiotics and anti-tumor drugs.

	**Gene**	**Synonyms**	**Proving reference**
1	*Abca1*	ABC1, HDLDT1, TGD	Hou et al., 2017
2	*Abca9*	–	Chen et al., 2009
3	*Abca13*	–	Hlavata et al., 2012
4	*Aldh1a1*	ALDH1, PUMB1, RALDH1	Jiang et al., 2016
5	*Aldh1l1*	FTHFD, 10-fTHF	Hartomo et al., 2015
6	*Amy1*	AMY1A	Mizuno et al., 2015
7	*Cd55*	DAF, CR, CROM, TC	Saygin et al., 2017
8	*Cd200*	MOX1, MOX2, MRC, OX-2	Jung et al., 2015b
9	*Cldn1*	ILVASC, SEMP1	Zhao et al., 2017
10	*Col3a1*	EDS4A	Januchowski et al., 2016
11	*Col6a2*	Collagen type VI alpha 2	Januchowski et al., 2016
12	*Cp*	Ceruloplasmin, ferroxidase	Chekhun et al., 2014
13	*Cyp7a1*	Cholesterol 7 alpha-monooxygenase	Eloranta and Kullak-Ublick, 2005
14	*Fgfr1*	BFGFR, CD331, CEK, FLG, H2, H3, H4, H5, N-SAM, FLT2, KAL2	Cole et al., 2010
15	*Gas6*	AXLLG, AXSF	Wang et al., 2016a
16	*Gstm3*	GST5	Black et al., 1990
17	*Grb10*	Growth factor receptor-bound protein 10	Roszak et al., 2013
18	*Igf1*	IGF-I, IGF1A, IGFI, somatomedin C	Kikuchi et al., 2015
19	*Igf2*	IGF-II, preptin, somatomedin A	Wozniak et al., 2015
20	*Il10*	CSIF, IL-10, IL10A, TGIF	Park et al., 2009
21	*Lyve1*	XLKD1, LYVE-1	Qin et al., 2011
22	*Nfatc2*	NF-ATP, NFAT1, NFATp	Griesmann et al., 2013
23	*Nt5e*	CALJA, CD73, eN, eNT	Loi et al., 2013
24	*Nts*	Neuromedin N, pro-neurotensin/neuromedin	Vias et al., 2007
25	*Pde4d*	DPDE3	Miklos et al., 2016
26	*Pdk4*	–	Zhang et al., 2016d
27	*Pf4*	CXCL4, SCYB4	Han et al., 1997
28	*Per2*	–	Mitchell and Engelbrecht, 2015
29	*Pon1*	Arylesterase 1, ESA	Aldonza et al., 2017
30	*Rasgrp3*	CalDAG-GEFIII, GRP3	Nagy et al., 2014
31	*Selp*	GRMP, CD62P, GMP140, PADGEM, PSEL	Zheng et al., 2013
32	*Serpinb2*	PAI2, PLANH2, HsT1201	Taoka et al., 2015
33	*Slco4a1*	SLC21A12, OATP-E, OATP4A1	Brenner et al., 2015
34	*Tal1*	TCL5, bHLHa17, SCL	Bernard et al., 1998
35	*Tnn*	TNW, TN-N, TN-W	Fukunaga-Kalabis et al., 2010
36	*Tubb1*	Class VI beta-tubulin	Li et al., 2014
37	*Vsig4*	Z39IG	Zhang et al., 2016c
38	*Wnt5a*	WNT-5A	Hung et al., 2014

*Genes symbols and synonyms are given in accordance to HGNC nomenclature*.

### Classification of genes contributing to tamra+ krebs-2 carcinoma cells malignancy with regard to their functional role in the formation of the tumorigenicity hallmarks

**Figure 1:**
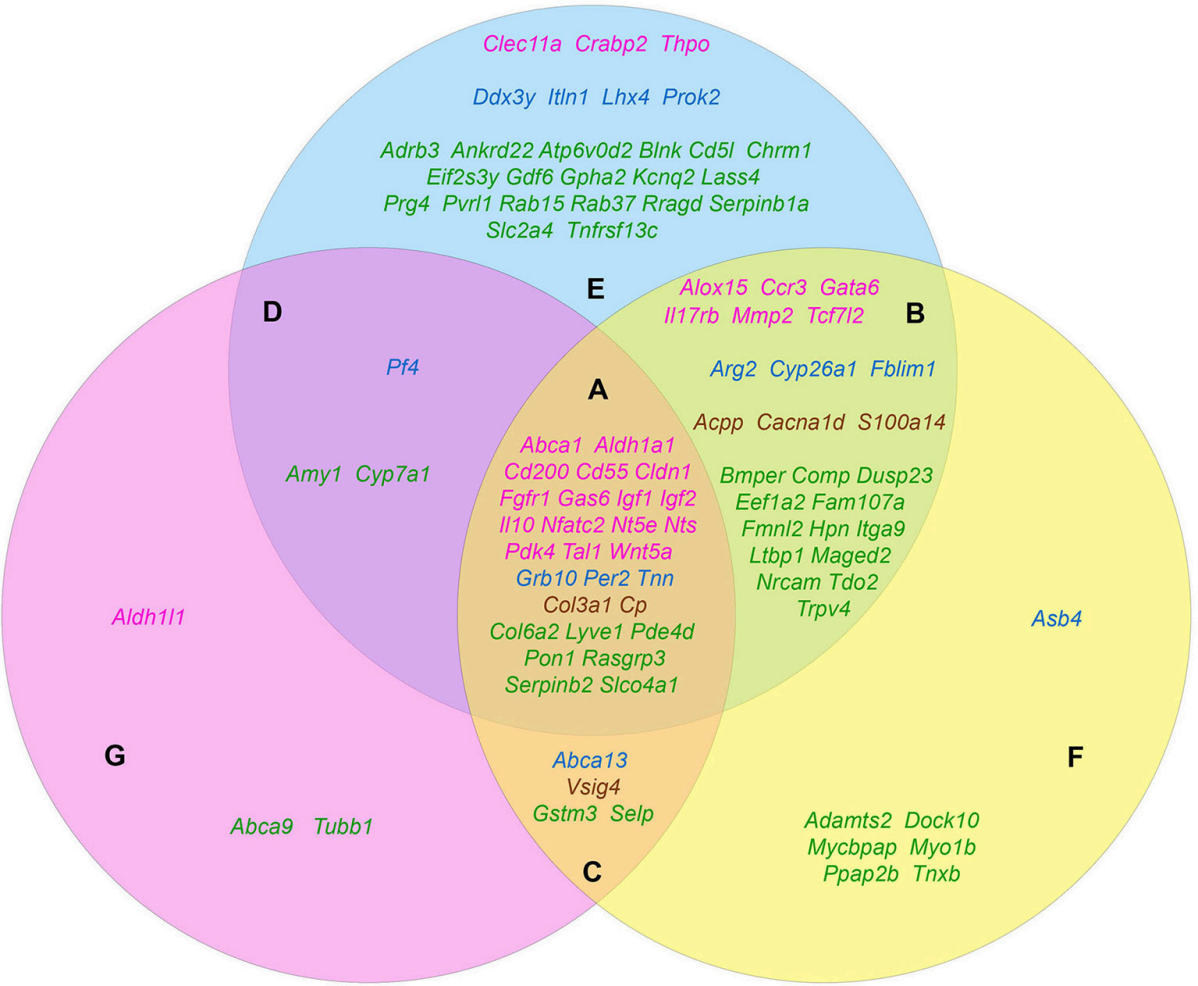
The distribution pattern of genes, overexpressed in TAMRA+ Krebs-2 carcinoma cells relative to TAMRA− cells, to functional groups. The blue area corresponds to the proliferative self-sufficiency, yellow, to invasiveness and metastasis; pink, to drug resistance. **(A–F)** Sections indicate the subgroups of the genes with regard to their multi-functionality: **(A)** the most multi-functional genes contributing to all three properties; **(B)** genes contributing to proliferative self-sufficiency and invasive growth/metastasis; **(C)** genes contributing to drug resistance and metastatic phenotype; **(D)** genes contributing to proliferative self-sufficiency and drug resistance; **(E)** genes contributing to proliferative self-sufficiency; **(F)** genes contributing to invasive growth and metastasis; **(G)** genes contributing to drug resistance. The genes symbols are indicated in different colors in accordance with their proven functional role in the formation of the stem or stem-like phenotype of normal pluri-/multipotent as well as tumor stem cells: pink denoting genes that are known markers of both normal pluri-/multipotent and tumor stem cells; blue, known markers of normal pluri-/multipotent stem cells; brown, known markers of tumor stem cells; green, genes for which their participation in the formation of stemness has not been proven at all.

The carried out data mining showed that out of 167 genes we tested, at least 96 belong to at least one of the three groups by their functional role in the formation of the tumorigenic phenotype. Herewith, all these genes in a completely natural way were dispensed into 7 additional groups. Group A (28 genes): *Abca1, Aldh1a1, Cd55, Cd200, Cldn1, Col3a1, Col6a2, Cp, Fgfr1, Gas6, Grb10, Igf1, Igf2, Il10, Lyve1, Nfatc2, Nt5e, Nts, Pde4d, Pdk4, Per2, Pon1, Rasgrp3, Serpinb2, Slco4a1, Tal1, Tnn, Wnt5a—*genes contributing to the formation of all three features. Group B (25 genes): *Acpp, Alox15, Arg2, Bmper, Cacna1d, Ccr3, Comp, Cyp26a1, Dusp23, Eef1a2, Fam107a, Fblim1, Fmnl2, Gata6, Hpn, Il17rb, Itga9, Ltbp1, Maged2, Mmp2, Nrcam, S100a14, Tcf7l2, Tdo2, Trpv4—*genes that simultaneously provide proliferative self-sufficiency and invasive growth/metastasis. Group C (4 genes): *Abca13, Gstm3, Selp, Vsig4—*genes that confer the drug resistance along with the metastatic phenotype. Group D (3 genes): *Amy1, Cyp7a1, Pf4—*genes responsible for proliferative self-sufficiency and drug resistance. Group E (26 genes): *Adrb3, Ankrd22, Atp6v0d2, Blnk, Cd5l, Chrm1, Clec11a, Crabp2, Ddx3y, Eif2s3y, Gdf6, Gpha2, Itln1, Kcnq2, Lass4, Lhx4, Prok2, Prg4, Pvrl1, Rab15, Rab37, Rragd, Serpinb1a, Slc2a4, Thpo, Tnfrsf13c*—genes responsible for proliferative self-sufficiency solely. Group F (7 genes): *Adamts2, Asb4, Dock10, Mycbpap, Myo1b, Ppap2b, Tnxb—*genes-inducers of invasive growth and metastasis. Group G (3 genes): *Abca9, Aldh1l1, Tubb1—*drug resistance genes (Figure 1).

Since the genes of the first four groups are “polyfunctional,” i.e., impact two or more properties simultaneously, it is logical to conclude that they contribute significantly greater (in comparison to the genes of the remaining three groups) to the formation of highly tumorigenic phenotype of the TAMRA+ cells. This makes them to be the most plausible candidates for the role of the main genetic markers of CSCs as well as malignancy itself. Moreover, the composed molecular-genetic “portrait,” emphasizing the differences of these cells from the bulk of tumor cells, gives additional reasons to believe that the main properties of tumor malignancy are determined precisely by CSCs.

### Formation and maintenance of stemness

Since the term “cancer stem cell” was introduced to designate a certain subpopulation of tumor cells on the basis of their phenotypic and functional similarity to normal pluri-/multipotent stem cells, it was initially assumed that there could be some common molecular-genetic mechanisms that provide such a similarity (Reya et al., 2001). Indeed, such stem cell-specific signaling pathways as, for example, *Wnt-, Notch-*, and *Shh-*dependent ones, have been demonstrated to be involved in development of various human and murine tumors (Ellisen et al., 1991; Henrique et al., 1997; Korinek et al., 1998; Chan et al., 1999; Wechsler-Reya and Scott, 1999, 2001; Zhang and Kalderon, 2001).

In this connection, we have considered it to be interesting to search the existing literature for evidences of the functional involvement of the identified genes-inducers of highly tumorigenic phenotype of the TAMRA+ cells in maintaining the stemness of normal pluri-/multipotent cells. In addition, we evaluated the involvement of these genes in the formation and maintenance of the stem-like phenotype of tumor cells.

Based on the screening results, 45 genes were assigned to the category of “stemness markers,” which makes up 46% of the analyzed and 27% of the total (167) genes differentially overexpressed in TAMRA+ cells of the Krebs-2 carcinoma. Herewith, more than half of these genes, namely 27 out of 45, are known to be implicated in maintenance and functional realization of stem properties of both tumor and normal pluripotent cells. However, four of these genes were included in this group with some reservations. Thus, for *Cd55* and *Il10*, no direct contribution to the formation or maintenance of stemness of normal pluri-/multipotent cells was proved, but the essential role in the realization of the reparative functions of mesenchymal and autologous-induced pluripotent stem cells by dint of the immunosuppressive action of the protein products of these genes was demonstrated (Ardianto et al., 2010; Liu et al., 2012; de Almeida et al., 2014; Lee et al., 2015b). The role of *Nts* in the formation of the pluripotent phenotype has been proved only in the case of the so-called induced pluripotent cells (Cai et al., 2015). And, finally, for *Crabp2* there was no direct evidence of its participation in the formation of stemness, but only demonstration of specific expression in normal human amniotic fluid-derived stem cells and in polycythemia vera-derived tumor stem cells (Steidl et al., 2005; Kim et al., 2010). In conjunction with its role in the metabolism of retinoic acids and their derivatives, this makes it to be attractive as a plausible marker of stemness for both tumor and normal stem cells.

Six more genes were identified as established markers of tumor-initiating stem cells. The remaining 12 genes were associated exclusively with normal pluripotent and multipotent stem cells, although, again, with some reservations. So, for *Abca13* only specific expression in early human embryonic stem cells, decreasing during consecutive passages has been shown (Barbet et al., 2012), while for *Arg2*, as well as for the above *Cd55* and *Il10*, its functional role is limited to the immunosuppressive effect necessary to overcome tissue-specific immunity by stem cells as it was shown for human pluripotent cells (Chen et al., 2015b). The results of the screening are summarized in Table 4.

**Table 4:** Genes showing elevated expression in TAMRA+ Krebs-2 carcinoma cells relative to TAMRA− cells, and participating in formation and maintenance of stem properties of tumorigenic as well as normal pluri-/multipotent stem cells.

	**Gene**	**Proof for the role in CSCs formation and maintenance**	**Proof for the role in normal pluri-/multi-potent stem cells formation and maintenance**
1	*Abca1*	Sun et al., 2015	Peeters et al., 2006
2	*Abca13*	N/C	Barbet et al., 2012
3	*Aldh1a1*	Yang et al., 2014c	Dey et al., 2015
4	*Aldh1l1*	Hartomo et al., 2015; Wang et al., 2016d	Foo and Dougherty, 2013
5	*Acpp*	Liu et al., 2014	N/C
6	*Alox15*	Chen et al., 2014d	Kinder et al., 2010
7	*Arg2*	N/C	Chen et al., 2015b
8	*Asb4*	N/C	Yang et al., 2014b
9	*Cacna1d*	Gerber et al., 2013	N/C
10	*Ccr3*	Long et al., 2012	Krathwohl, 2004
11	*Cd55*	Saygin et al., 2017	Ardianto et al., 2010
12	*Cd200*	Jung et al., 2015b	Wang et al., 2014
13	*Cldn1*	Mahati et al., 2017a	Zinner et al., 2013
14	*Clec11a*	Hiraoka, 2008	Hiraoka et al., 2001
15	*Col3a1*	Januchowski et al., 2016	N/C
16	*Cp*	Tye et al., 2008; Brandi et al., 2016	N/C
17	*Crabp2*	Kim et al., 2010	Steidl et al., 2005
18	*Cyp26a1*	N/C	Assou et al., 2007
19	*Ddx3y*	Rosinski et al., 2008	Kotov et al., 2017
20	*Fblim1*	N/C	Xiao et al., 2012
21	*Fgfr1*	Ji et al., 2016	Coutu et al., 2011
22	*Gas6*	Jin et al., 2017b	Gely-Pernot et al., 2012
23	*Gata6*	Whissell et al., 2014	Kubo et al., 2009
24	*Grb10*	N/C	Li et al., 2017a
25	*Igf1*	Bu et al., 2014	Li et al., 2010b
26	*Igf2*	Tominaga et al., 2017	Bendall et al., 2007
27	*Il10*	Tuccitto et al., 2016	Liu et al., 2012; de Almeida et al., 2014; Lee et al., 2015b
28	*Il17rb*	Bie et al., 2016	Bie et al., 2017
29	*Itln1*	N/C	Zhao et al., 2015
30	*Lhx4*	N/C	Chen et al., 2005
31	*Mmp2*	Sun et al., 2013; An et al., 2015	Huang et al., 2011
32	*Nfatc2*	Perotti et al., 2016	Kiani et al., 2004
33	*Nt5e*	Katsuta et al., 2016	Corradetti et al., 2013
34	*Nts*	Zhou et al., 2014	Cai et al., 2015
35	*Pdk4*	Song et al., 2015	Takubo et al., 2013
36	*Per2*	N/C	Boucher et al., 2016
37	*Pf4*	N/C	Han et al., 1997; Calaminus et al., 2012; Chen et al., 2014b
38	*Prok2*	N/C	LeCouter et al., 2004
39	*S100a14*	Leth-Larsen et al., 2012; Ko et al., 2013	N/C
40	*Tal1*	Gerby et al., 2016	Baharvand et al., 2006; Souroullas et al., 2009
41	*Tcf7l2*	Chen et al., 2015a	Quan et al., 2016
42	*Thpo*	Chou et al., 2012	Kohlscheen et al., 2015
43	*Tnn*	N/C	Tucker et al., 2013
44	*Vsig4*	Zhang et al., 2016c	N/C
45	*Wnt5a*	Zhou et al., 2017	Hao et al., 2006

### Malignancy and pluripotency: looking for difference

Identification of such an entity as a CSC has allowed to apply the principles of organogenesis to the development of tumors. From this point of view, the tumor is considered to be an aberrant organ, developing from a tumor cell possessing an infinite proliferative potential and a poorly differentiated stem-like phenotype (Reya et al., 2001). This approach implies the existence of functional analogies between normal stem cells involved in embryogenesis and tumor stem cells. Taking into account the functional purpose of normal pluri-/multipotent cells, their basic physiological properties can be deduced. First, it is obvious that the stem cell must possess a certain degree of proliferative autonomy and increased survival abilities to realize the function of the population self-maintenance. Second, the stem cell must evince active migratory and immunosuppressive functions, as well as the multiple tissue adherence to realize its genesis/reparative/regenerative functions. And third, the stem cell must have a well developed system of detoxification and resistance to xenobiotics, to keep the genome of both its own and the population as a whole intact. i.e., the attributes of stemness and the ones of malignancy, which we defined above, are the same, at least in the first approximation, and, respectively, the molecular-genetic mechanisms that determine these two characteristics can overlap to a significant degree.

Based on Table 4 and Figure 1 data, it can be noted that 21 of the 45 stemness marker genes got into Group A, which includes 28 genes that are most important for the formation of TAMRA+ cells malignancy. That is, this group substantially (75%) consists of the genes essential for the formation and maintenance of stem properties. At this, only two genes are identified as indicators of the stem-like phenotype of tumor cells, while the remaining 19 are necessary for the functioning of normal pluri-/multipotent stem cells. Another 12 genes were included into group B consisting of a total of 25 genes, while the rest were more or less evenly distributed over the remaining five groups.

Thus, the identity of genes determining the malignant properties of tumor-initiating cells and the stem properties of normal pluri-/multipotent stem cells has been revealed. Molecular-genetic identity of tumor-initiating and normal stem cells, as well as their morphophysiological one, gave us a reason to presume the identity of the very properties of malignancy and pluripotency themselves, that can be also designated as the properties of “independent behavior.” Up to the day, a significant number of evidences confirming the presumed behavioral identity of both types of cells has been presented. Thus, for example, it had been shown that transplantation of human embryonic stem cells, as well as of diploid and aneuploid pluripotent ones can lead to the development of tumors, most commonly identified as benign teratomas or malignant teratocarcinomas (Blum and Benvenisty, 2008, 2009). This property is postulated to be the hallmark of all pluripotent stem cell types, which demonstrates their potential to differentiate in all tissue types (reviewed in Dressel, 2011). On the other hand, classical experiments on the inoculation of teratocarcinomas cells into mouse embryos at the early stages of development have shown that, getting into the “right” conditions, malignant cells can differentiate into normal tissue, resulting in the development of a normal mosaic organism (Martin and Evans, 1975; Mintz and Illmensee, 1975; Illmensee and Mintz, 1976).

In other words, all these facts could mean that malignancy and stemness/pluripotency are one and the same entity, and the way this entity could be realized—malignancy or normal stemness—depends on the cellular microenvironment that provides the mentioned “right” location and conditions. And it is the stem cell niche that is apparently to be the appropriate location with appropriate conditions.

Initially, the term “stem cell niche” was proposed by Schofield in 1978 to describe a hypothetical cellular structure that provides conditions for the existence of a stem cell in which it is able to maintain its basic properties of self-renewal and maintenance of an undifferentiated or poorly differentiated state (Schofield, 1978). In its contemporary meaning, the role of the stem niche is dedicated to two basic functions. The first is to maintain the population of stem cells at a certain level by balancing pro-mitogenic and anti-mitogenic signals and providing a specific microenvironment necessary to maintain the undifferentiated state of stem cells (Schofield, 1983; Lin, 2002; Ohlstein et al., 2004; reviewed in Li and Neaves, 2006). The second is to act as a kind of “Maxwell's demon,” allowing niche exit to committed precursor cells, but not stem ones (reviewed in Marthiens et al., 2010). The last function has its reverse. The implication is that if a stem cell leaves the niche for any reason, it must either go back—the so-called “homing” known for hematopoietic stem cells, which can leave the stem niche for a while and then return (Whetton and Graham, 1999), or lose stemness and switch to a committed state, which, finally, ends with differentiation (Voog and Jones, 2010; O'Brien and Bilder, 2013). Simply stated, stem cells could not exist outside the stem cell niche. The main, as well as the only difference between CSCs and normal stem cells which is, in fact, the property of malignancy itself, is the ability to form and maintain stem/pluripotent properties outside a specific niche. This property comprises the defiance to morphogenetic signals from normal cellular and stromal environment and, as a consequence, the ability to form the tumorous stroma as well as the tumor itself in any tissue of the organism independently on the local environmental conditions.

Summarizing the section it should be said that the search for mechanisms providing such “independent behavior” of the CSCs is the principal priority in fundamental molecular oncology for now.

## Cancer stem cells: ultima ratio of tumors?

The hypothesis of “dynamic stemness” presumes the inducibility of stem-like phenotype in some subpopulation of “committed” tumor cells. It seems to be logical that such an induction and the following *de novo* appearance of CSCs occurs rather due to certain changes in cellular humoral or stromal environment. Thus, revealing the genes responsible for the stemness of TAMRA+ cells of the Krebs-2 carcinoma allows, in addition to the above, to deduce both the causes and mechanisms of induction of the stem-like phenotype in some part of the tumor cells.

### “Generalized cellular stress” as an activator of “stemness genes”

**Figure 2:**
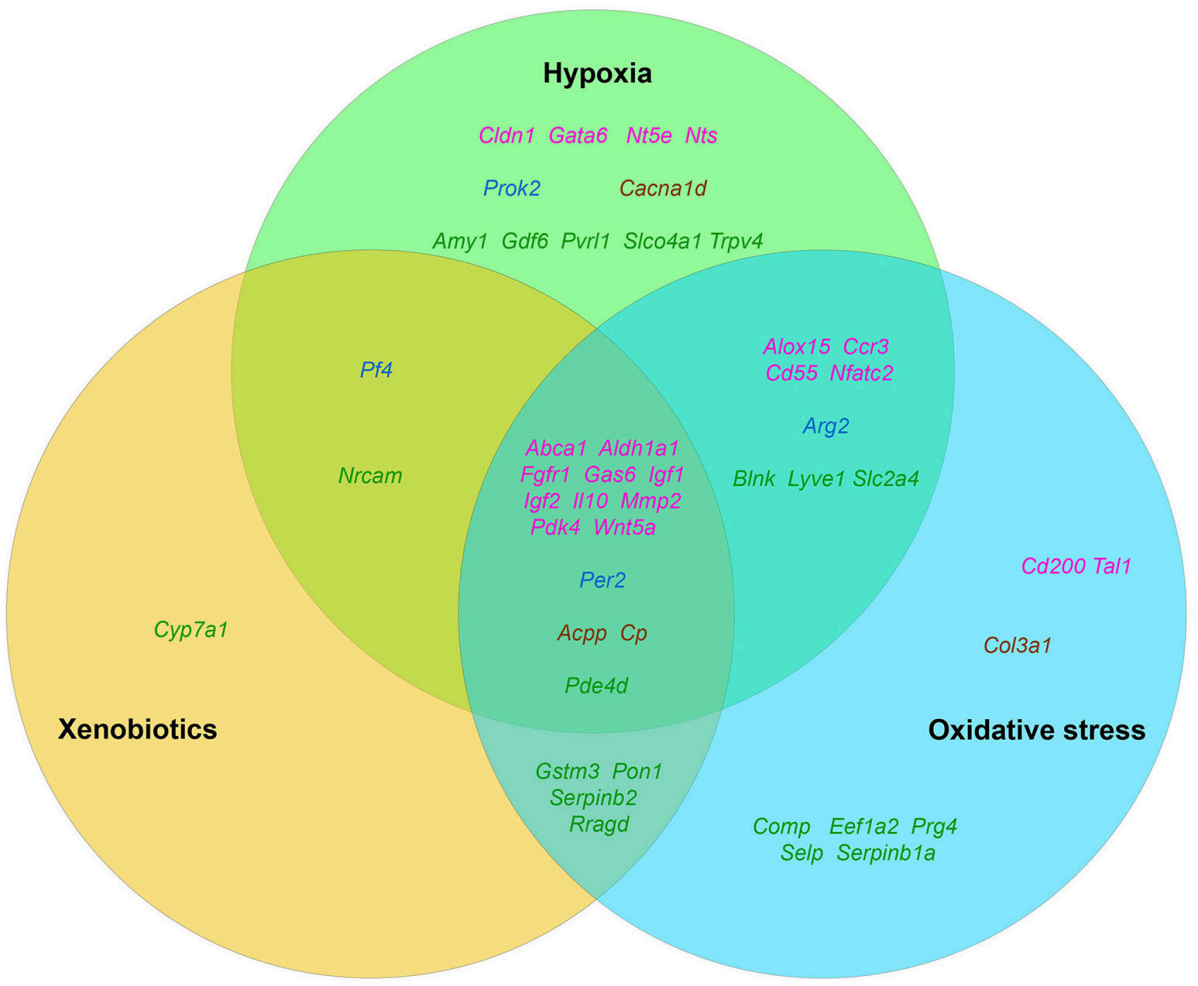
The distribution pattern of genes, overexpressed in TAMRA+ Krebs-2 carcinoma cells relative to TAMRA− cells, with regard to their activation in response to appropriate stimulus. The green area corresponds to hypoxia, blue–to oxidative stress, orange–to xenobiotics. The genes symbols are indicated in different colors in accordance with their proven functional role in the formation of the stem or stem-like phenotype of normal pluri-/multipotent as well as tumor stem cells: pink denoting genes that are known markers of both normal pluri-/multipotent and tumor stem cells; blue–known markers of normal pluri-/multipotent stem cells; brown–known markers of tumor stem cells; green–genes for which their participation in the formation of stemness has not been proven at all.

It is well known that tumor growth and development is always accompanied by a number of stress factors. The first of them is the formation of hypoxia foci (Moulder and Rockwell, 1987; reviewed in Bertout et al., 2008). The second one is the oxidative stress, which develops due to various inflammatory and immune reactions (reviewed in Murr et al., 1999; Laviano et al., 2007). And, finally, an increased level of endogenous xenobiotics, such as, for example, kynurenine (Kurz et al., 2011), that are able to activate both AhR (Poormasjedi-Meibod et al., 2016) and other xenosensors. Accordingly, we decided to check the published data in order to find out how much these stress factors are capable of activating the stemness of tumor cells in general as well as the expression of selected “stemness genes” in particular (Figure 2).

The fact that hypoxia is a strong stimulus that enhances the aggressive behavior of tumors had been known for a long time (reviewed in Bertout et al., 2008). More recent studies have shown that hypoxia is mandatory for the existence of normal embryonic and other pluri-/multipotent stem cells (Mohyeldin et al., 2010; López-Iglesias et al., 2015; Hammoud et al., 2016), and induces the stem-like phenotype in prostate cancer (Bae et al., 2016), human lung cancer (Iida et al., 2012) and other types of tumors (reviewed in Li and Rich, 2010; Seo et al., 2016). The existing data analysis has revealed that 35 of the 96 genes we have identified as essential for stemness are activated, one way or another, in conditions of local or systemic hypoxia (Table 5, Figure 2).

**Table 5:** The results of the analysis of published data on activating effect of the factors of generalized cellular stress for the tested genes that provide a malignant/pluripotent phenotype of the Krebs-2 CSCs.

**Activated genes**	**Components of generalized cellular stress**		
	**Hypoxia**	**Oxidative stress**	**Xenobiotics**
48 genes	35 genes	34 genes	21 genes
*Abca1*	Plösch et al., 2010	Davies et al., 2015	Ma and Liu, 2012
*Acpp*	Liu et al., 2014	Obianime and Roberts, 2009	Obianime and Roberts, 2009
*Aldh1a1*	Hough and Piatigorsky, 2004	Strzalka-Mrozik et al., 2013	Hough and Piatigorsky, 2004
*Alox15*	Lundqvist et al., 2016	Jung et al., 2015a	N/C
*Amy1*	Jam et al., 1978	N/C	N/C
*Arg2*	Chen et al., 2014a	Touyz, 2014	N/C
*Blnk*	Han et al., 2001	Han et al., 2001	N/C
*Cacna1d*	Li et al., 2015	N/C	N/C
*Ccr3*	Ricciardi et al., 2008	Michalec et al., 2002	N/C
*Cd200*	N/C	Gupta et al., 2014	N/C
*Cd55*	Botto et al., 2008	Iborra et al., 2003	N/C
*Cldn1*	Brown et al., 2003	N/C	N/C
*Col3a1*	N/C	Luna et al., 2009	N/C
*Comp*	N/C	Wahba et al., 2016	N/C
*Cp*	Martin et al., 2005	Dzugkoeva et al., 2016	Auslander et al., 2008
*Cyp7a1*	N/C	N/C	Lambert et al., 2009
*Eef1a2*	N/C	Chen et al., 2000	N/C
*Fgfr1*	Ganat et al., 2002; Mouillet et al., 2013	Alizadeh et al., 2001	Łazarenkow et al., 2017
*Gas6*	Mishra et al., 2012	Tsai et al., 2016	Bruchova et al., 2010
*Gata6*	Hsiao et al., 2015	N/C	N/C
*Gdf6*	Li et al., 2013	N/C	N/C
*Gstm3*	N/C	Gibson et al., 2014	Li et al., 2016b
*Igf1*	Yang et al., 2015	Jiao et al., 2015a	Wohlfahrt-Veje et al., 2014
*Igf2*	Jögi et al., 2004	Yang et al., 2014a	Wang et al., 2011
*Il10*	Xu et al., 2016	Joseph Martin and Evan Prince, 2017	Pacheco et al., 2001; Khalil et al., 2010
*Lyve1*	Chaudary et al., 2011	Jiao et al., 2015a	N/C
*Mmp2*	Slevin et al., 2009	Liu et al., 2017	Kamaraj et al., 2010
*Nfatc2*	Wang et al., 2016b	Nomura et al., 2011	N/C
*Nrcam*	Slevin et al., 2009	N/C	Gato et al., 2012
*Nt5e*	Fu and Davies, 2015	N/C	N/C
*Nts*	Shen and Wang, 1998	N/C	N/C
*Pde4d*	Pullamsetti et al., 2013	Kim et al., 2017	Yeo et al., 2017
*Pdk4*	Van Thienen et al., 2017	Lake et al., 2016	Luckhurst et al., 2011
*Per2*	Peek et al., 2017	Davis et al., 2017	Manzella et al., 2013
*Pf4*	Shen and Wang, 1994	N/C	Sithu et al., 2010
*Pon1*	N/C	Desai et al., 2014	Gouédard et al., 2004
*Prg4*	N/C	Lee et al., 2015a	N/C
*Prok2*	LeCouter et al., 2003	N/C	N/C
*Pvrl1*	Friedman et al., 2012	N/C	N/C
*Rragd*	N/C	Sasaki et al., 2012	Sasaki et al., 2012
*Selp*	N/C	Takano et al., 2002	N/C
*Serpinb1a*	N/C	Frühbeck et al., 2010	N/C
*Serpinb2*	N/C	Leeuwen et al., 2006; Vo et al., 2011	Brauze et al., 2017
*Slc2a4*	Royer et al., 2000	Li et al., 2010a	N/C
*Slco4a1*	Applebaum et al., 2016	N/C	N/C
*Tal1*	N/C	Ogino et al., 2014	N/C
*Trpv4*	Wu et al., 2017	N/C	N/C
*Wnt5a*	Chen et al., 2006	Andersson-Sjöland et al., 2016	Hrubá et al., 2011

Data on the role of oxidative stress in regulation of tumor cells stemness are in general quite contradictory. Numerous studies confirm suppression of the stem-like phenotype of tumor cells under oxidative stress conditions in, for example, *in vitro* experiments with SUM159 breast cancer cells and pancreatic CSCs of various origin (Cipak et al., 2010; Ma et al., 2017). On the other hand there are numerous quite convincing direct evidences of stemness induction in response to oxidative stress, as, for example, in MCF7 and ZR751 breast cancer cells (Gopal et al., 2016) or in lung cancer cells (Saijo et al., 2016) as well as in a number of other *in vitro* models (reviewed in Dayem et al., 2010). We found the evidences of oxidative stress activating effect for 34 genes of our list (Table 5, Figure 2).

Finally, we have found data, albeit not numerous, confirming that xenobiotics are also able to induce the stemness of tumor cells. This was shown, for example, for human bronchial epithelial cells (Liu et al., 2016c) and SUM149 inflammatory breast cancer cells (Stanford et al., 2016). As well, xenobiotics turned out to activate the expression of 21 out of 96 genes of stemness of TAMRA+ cells of the Krebs-2 tumor (Table 5, Figure 2).

Thus, the datamining analysis showed that 48 of the 96 genes we designated as potentially important for the formation of the poorly differentiated/stem-like phenotype of tumor cells are activated in response to at least 1 of 3 stress stimuli–hypoxia, oxidative stress, or xenobiotics. Moreover, 14 genes (*Aldh1a1, Abca1, Igf1, Igf2, Il10, Gas6, Fgfr1, Wnt5a, Pdk4, Per2, Cp, Pde4d, Mmp2, Acpp*) respond with increased expression to all 3 stimuli. It is easy to note that 12 of these 14 genes belong to group A (Figure 1), which contains genes most significant for maintaining stemness/malignancy. Moreover, visual representation of these results in Figure 2 signifies the multiplicity of inducing agents for the majority of stemness-specific genes (pink, blue, and brown denoted ones).

It is known that none of the mentioned stress stimuli exist separately *in vivo*, instead they are always inextricably linked to each other. So, hypoxia, as well as the presence of xenobiotics, lead to oxidative stress (Netzer et al., 2015; Pizzino et al., 2017). On the other hand, oxidative stress leads to a corruption in metabolism that, in turn, causes the formation of various endogenous xenobiotics such as kynurenine (Ramírez-Ortega et al., 2017; Wigner et al., 2018) or tryptamine-4,5-dione (Jiang et al., 1999; Suga et al., 2017). Therefore, we decided to combine these three stress factors into the single concept of “generalized cellular stress.”

### Roads to rome: molecular mechanisms of stemness induction

It is quite obvious that the fact that we have not found any data on the impact of generalized cellular stress on the remaining 48 genes does not mean that there really is no such an influence. Our hypothesis on the role of stress in the activation of stemness could be to some extent supported by data on the presence of regulatory elements that provide the binding of transcription factors and transcriptional activation of these genes in response to factors of generalized cellular stress. To conduct such analysis, we used the open web resource “Enrichr: interactive and collaborative HTML5 gene list enrichment analysis tool”: http://amp.pharm.mssm.edu/Enrichr/ (Chen et al., 2013; Kuleshov et al., 2016). Databases of this tool contain an excessive compilation of a huge number of results obtained in the ChIP-Seq (Chromatin ImmunoPrecipitation-Sequencing) experiments. It allows to use the tool not only to determine the degree of sampling enrichment by the criterion of the presence of functional binding sites for certain transcription factors, but also in principle to determine the presence of such sites in the subject genes. So we used the “ChEA 2016” section of the tool to test all 96 “stemness genes” for the presence of binding sites for transcription factors established by experiments on ChIP-Seq analysis. One of the main outcomes of this analysis was that the 72 genes from our list contain binding sites for the SOX2 transcription factor, 59–OCT4/POU5F1, 54–NANOG, 45–KLF4, and 52–c-MYC (Table 6). In fact, only 7 genes out of 96 (*Lyve1, Il17rb, Fam107a, Nrcam, Vsig4, Pf4, Amy1, Eif2s3y*) contained no binding sites for any of the listed factors.

**Table 6:** Results of “ChEA 2016” analysis for 96 ≪stemness genes≫ showing elevated expression in TAMRA+ Krebs-2 carcinoma cells relative to TAMRA− cells, with regard to enrichment with SOX2/OCT4/POU5F1/NANOG/KLF4/c-MYC binding sites.

**TrF**	**Overlap**	***P*-value**	**Genes**
SOX2	21/2564	0.009245	*Arg2; Crabp2; Ankrd22; Pde4d; Gata6; Cldn1; Dusp23; Myo1b; Nt5e; Fmnl2; Rab15; Ppap2b; Aldh1a1; Rragd; Pdk4; Maged2; Asb4; S100a14; Gas6; Cd55; Fgfr1*
SOX2	17/1991	0.013342	*Il10; Gstm3; Tcf7l2; Fblim1; Wnt5a; Alox15; Clec11a; Igf1; Tnfrsf13c; Nt5e; Adrb3; Ppap2b; Kcnq2; Rragd; Blnk; Asb4; Fgfr1*
SOX2	16/2000	0.028265	*Il10; Serpinb2; Itln1; Nfatc2; Prg4; Dock10; Selp; Per2; Dusp23; Col3a1; Myo1b; Fmnl2; Tnn; Kcnq2; Lhx4; Cd55*
SOX2	8/1278	0.270262	*Per2; Tcf7l2; Tal1; Rab15; Rragd; Maged2; Pvrl1; Fgfr1*
SOX2	14/2000	0.096132	*Gstm3; Fblim1; Clec11a; Gata6; Itln1; Nfatc2; Cyp7a1; Per2; Lass4; Aldh1a1;Pdk4;Blnk; Gas6; Atp6v0d2*
SOX2	14/2000	0.096132	*Il10; Abca1; Cd5l; Abca9; Cacna1d; Igf1; Cp; Thpo; Rab37; Slco4a1; Tubb1; Maged2; Atp6v0d2; Cd200*
SOX2	13/2000	0.160414	*Il10; Tcf7l2; Aldh1l1; Arg2; Wnt5a; Nfatc2; Cacna1d; Cp; Tal1; Grb10; Lhx4; Acpp; Cd55*
SOX2	5/863	0.399596	*Per2; Gata6; Gdf6; Ltbp1; Itga9*
SOX2	19/3319	0.235257	*Il10; Gstm3; Tcf7l2; Fblim1; Alox15; Clec11a; Wnt5a; Igf1; Tnfrsf13c; Nt5e; Adrb3; Slco4a1; Ppap2b; Kcnq2; Rragd; Blnk; Asb4; Fgfr1; Itga9*
SOX2	2/497	0.692959	*Per2; Myo1b*
SOX2	3/785	0.732379	*Il10; Per2; Ppap2b*
SOX2	19/3420	0.278900	*Pde4d; Hpn; Nfatc2; Cacna1d; Gdf6; Abca13; Ltbp1; Rasgrp3; Dock10; Fmnl2; Bmper; Tnn; Slco4a1; Rab37; Kcnq2; Aldh1a1; Lhx4; Pvrl1; Ccr3*
SOX2	10/2000	0.495379	*Aldh1l1; Adamts2; Bmper; Rab15; Eef1a2; Hpn; Tubb1; Abca9; Gpha2; Pvrl1*
OCT4	20/2000	0.001144	*Il10; Serpinb2; Wnt5a; Itln1; Prg4; Igf1; Nts; Abca13; Dock10; Selp; Per2; Dusp23; Col3a1; Myo1b; Adamts2; Tnn; Col6a2; Grb10; Lhx4; Cd55*
OCT4	16/2000	0.028265	*Il10; Serpinb2; Itln1; Nfatc2; Prg4; Dock10; Selp; Per2; Dusp23; Col3a1; Myo1b; Fmnl2; Tnn; Kcnq2; Lhx4; Cd55*
OCT4	13/1992	0.157087	*Il10; Gstm3; Tcf7l2; Wnt5a; Alox15; Nfatc2; Nt5e; Adrb3; Kcnq2; Rragd; Asb4; Pvrl1; Fgfr1*
OCT4	13/2000	0.160414	*Tnxb; Cacna1d; Slc2a4; Comp; Adamts2; Tdo2; Adrb3; Slco4a1; Trpv4; Kcnq2; Rragd; Pvrl1; Itga9*
OCT4	7/2000	0.856909	*Tcf7l2; Eef1a2; Hpn; Grb10; Igf1; Acpp; Rasgrp3*
POU5F1	12/1550	0.067193	*Adamts2; Crabp2; Bmper; Thpo; Adrb3; Pde4d; Rragd; Pdk4; Cacna1d; Mycbpap; Asb4; Fgfr1*
POU5F1	6/622	0.078803	*Nt5e; Tal1; Mmp2; Gata6; Maged2; Fgfr1*
POU5F1	12/2109	0.311132	*Il10; Myo1b; Nt5e; Fmnl2; Adrb3; Fblim1; Tal1; Ppap2b; Cacna1d; Maged2; Asb4; Igf1*
POU5F1	3/753	0.705916	*Il10; Per2; Cyp26a1*
POU5F1	1/567	0.937187	*Slco4a1*
POU5F1	1/555	0.933343	*Prok2*
POU5F1	2/559	0.753373	*Cyp26a1; Abca13*
POU5F1	18/4232	0.755639	*Il10; Gstm3; Tcf7l2; Fblim1; Alox15; Wnt5a; Nfatc2; Tnfrsf13c; Nt5e; Adrb3; Slco4a1; Ppap2b; Kcnq2; Rragd; Asb4; Pvrl1; Fgfr1; Itga9*
NANOG	15/1989	0.051785	*Tcf7l2; Wnt5a; Igf2; Igf1; Tnfrsf13c; Serpinb1a; Nt5e; Adrb3; Ppap2b; Kcnq2; Rragd; Blnk; Asb4; Pvrl1; Fgfr1*
NANOG	14/2000	0.096132	*Il10; Serpinb2; Itln1; Nfatc2; Prg4; Dock10; Selp; Dusp23; Col3a1; Myo1b; Fmnl2; Tnn; Lhx4; Cd55*
NANOG	19/3052	0.137575	*Tcf7l2; Wnt5a; Igf2; Igf1; Tnfrsf13c; Ltbp1; Serpinb1a; Nt5e; Adrb3; Rab15; Slco4a1; Ppap2b; Kcnq2; Rragd; Blnk; Asb4; Pvrl1; Itga9; Fgfr1*
NANOG	13/2000	0.160414	*Il10; Tcf7l2; Aldh1l1; Arg2; Wnt5a; Nfatc2; Cacna1d; Cp; Tal1; Grb10; Lhx4; Acpp; Cd55*
NANOG	9/1686	0.420900	*Tcf7l2; Tdo2; Rab15; Tal1; Mmp2; Gata6; Lhx4; Cldn1; Fgfr1*
NANOG	5/840	0.377515	*Clec11a; Blnk; Igf1; Acpp; Itga9*
NANOG	16/3520	0.636674	*Pde4d; Igf1; Cp; Lass4; Col3a1; Myo1b; Nt5e; Bmper; Adrb3; Rab37; Ppap2b; Pdk4; Grb10; Pvrl1; Fgfr1; Itga9*
NANOG	11/1908	0.307350	*Tcf7l2; Serpinb1a; Adrb3; Fblim1; Ppap2b; Rragd; Igf2; Blnk; Igf1; Itga9; Fgfr1*
NANOG	1/344	0.811672	*Igf2*
NANOG	2/542	0.737895	*Serpinb1a; Dusp23*
NANOG	3/1232	0.940192	*Slco4a1; Blnk; Igf1*
NANOG	8/2000	0.756029	*Tcf7l2; Dusp23; Arg2; Tal1; Prok2; Igf1; Gpha2; Ccr3*
KLF4	12/1211	0.013033	*Col3a1; Adamts2; Nt5e; Fblim1; Ankrd22; Gata6; Prok2; Slc2a4; Ltbp1; Cd200; Ccr3; Itga9*
KLF4	16/2000	0.028265	*Il10; Serpinb2; Itln1; Nfatc2; Prg4; Dock10; Selp; Per2; Dusp23; Col3a1; Myo1b; Fmnl2; Tnn; Slco4a1; Lhx4; Cd55*
KLF4	13/2000	0.160414	*Il10; Gstm3; Tcf7l2; Chrm1; Fblim1; Gata6; Cyp26a1; Myo1b; Rab37; Slco4a1; Ppap2b; Pvrl1; Cd55*
KLF4	8/1502	0.433701	*Cyp26a1; Bmper; Tal1; Ppap2b; Gata6; Igf2; S100a14; Cd55*
KLF4	6/2444	0.982047	*Per2; Lass4; Bmper; Col6a2; Tnfrsf13c; Acpp*
KLF4	7/1700	0.717649	*Myo1b; Lass4; Slco4a1; Igf2; Nfatc2; Pvrl1; Cd55*
KLF4	10/2000	0.495379	*Comp; Myo1b; Lass4; Slco4a1; Tal1; Pon1; Gata6; Grb10; Lhx4; Pvrl1*
CMYC	16/2000	0.028265	*Il10; Serpinb2; Itln1; Nfatc2; Prg4; Dock10; Selp; Per2; Dusp23; Col3a1; Myo1b; Fmnl2; Tnn; Kcnq2; Lhx4; Cd55*
MYC	14/2000	0.096132	*Ddx3y; Tnfrsf13c; Gdf6; Rasgrp3; Selp; Cyp26a1; Adamts2; Tal1; Rragd; Blnk; Asb4; Gas6; Pvrl1; Fgfr1*
MYC	16/2979	0.354148	*Gstm3; Tcf7l2; Tnxb; Crabp2; Mmp2; Wnt5a; Gata6; Igf2; Igf1; Ltbp1; Adamts2; Thpo; Rab15; Asb4; Lhx4; Cd55*
MYC	3/797	0.741816	*Blnk; Nfatc2; Tnfrsf13c*
MYC	2/3413	1.000000	*Per2; Adrb3*
MYC	6/3868	0.999934	*Tcf7l2; Clec11a; Wnt5a; Grb10; Gata6; Gdf6*
MYC	2/1458	0.994109	*Lass4; Fgfr1*
MYC	2/746	0.877854	*Rab37; Prok2*
MYC	4/1406	0.912685	*Mmp2; Ppap2b; Prok2; Tnfrsf13c*
MYC	11/2000	0.364090	*Ddx3y; Myo1b; Arg2; Slco4a1; Ankrd22; Hpn; Igf2; Grb10; Prg4; Igf1; Rasgrp3*

SOX2, OCT4/POU5F1, Nanog, KLF4 and c-Myc are known to be the five main transcription factors forming the transcriptional profile of stem cells. Activation of these factors is sufficient for reprogramming a normal somatic cell into a pluripotent/multipotent stem cell, as was first demonstrated on mouse embryonic and adult fibroblast cultures (Takahashi and Yamanaka, 2006; reviewed in Heng et al., 2010). These transcription factors are also shown to be activated under conditions of hypoxia (Li and Rich, 2010; Mathieu et al., 2011; Iida et al., 2012; Bae et al., 2016), oxidative stress (Cullingford et al., 2008; Kang et al., 2009; Kim et al., 2013; Chang et al., 2014; Balvan et al., 2015; Saijo et al., 2016) and in the presence of xenobiotics (Jang et al., 2014; Liu et al., 2016c; Stanford et al., 2016). Thus, the mechanism of formation of the tumor cells stemness can be proposed. This mechanism implies the activation of these key factors under conditions of generalized cellular stress that, in turn, leads to increased expression of specific targets, which probably include also the genes providing the stem-like phenotype of Krebs-2 cells.

In addition, we have decided to check for the possibility of an alternative mechanism of the “stem-genes” activation under conditions of generalized cellular stress independent of SOX2/OCT4/Nanog/KLF4/c-Myc pathway.

The main transcription factors that provide a cellular response to hypoxia are the proteins of the HIF family (hypoxia-inducible factor) (reviewed in Peet et al., 2017). However, the factors such as NFkB, CREB, AP-1, Egr-1, NF-IL6/C/EBPβ, RTEF-1, GATA2, STAT5, ETS1 (reviewed in Cummins and Taylor, 2005) as well as RUNX1 (Lee et al., 2017a) also take a direct part in the regulation of transcription under hypoxia/anoxia. ChIP-Seq enrichment analysis has revealed that 92 of 96 genes contain binding sites for at least one of these transcription factors with the following distribution: CREB1–25 genes, RELA (NFκB)−9 genes, cJUN (AP-1)−31 genes, MAF (AP-1)−15 genes, EGR1–42 genes, C/EBPβ-59 genes, ETS1–11 genes, STAT5–4 genes, GATA2–33 genes, RTEF-1/TEAD4–15 genes, RUNX1/AML1–45 genes (data not shown).

In addition, 88 genes contain a binding site(s) for such xenosensors or their intermediators, as PPARα/δ/γ (58 genes), NFE2L2/NRF2 (14 genes), AHR (6 genes), NR1I2/PXR (9 genes), FOXO1/3 (17 genes) (Klotz and Steinbrenner, 2017), MITF (25 genes) (Huang et al., 2013), EGR1 (42 genes) (Thiel and Cibelli, 2002; Sullivan et al., 2012), as well as for androgen receptor (AR) (55 genes). The last one had been shown to be activated not only by steroid hormones, but also by various xenobiotics, including endogenous ones as well (Araki et al., 2005) (data not shown).

Compared with other components of generalized cellular stress, oxidative stress activates the widest range of transcription factors, among them NFE2L2/NRF2, NFκB, cJUN, MAF, FOXO1/3, STAT1/3, ELK1, MEF2A (Zhang et al., 2016b; Klotz and Steinbrenner, 2017; Nemmiche, 2017; Sies et al., 2017), FLI1 and HOXB4 (Monzen et al., 2011), C/EBPα (Xu et al., 2009; Puri et al., 2012), C/EBPδ (Hour et al., 2010; Banerjee et al., 2016), MYB (Wan et al., 2005), GATA3 (Li et al., 2017b), and IRF8 (Li et al., 2017c; Sakai et al., 2017). It turned out that all 96 genes of our list contain site(s) for at least one of these transcription factors. The most represented factor was FLI1 (56 genes), followed by GATA3 (53 genes) and STAT3 (52 genes). Another 9 factors composed the group of average representation: cJUN−31 genes, IRF8–22 genes, C/EBPα-22 genes, C/EBPδ-21 genes, MYB−19 genes, MAF−15 genes, NFE2L2/NRF2–14 genes, FOXO1 and STAT1–12 genes for each. The remaining 5 factors were low-represented: NFκB/RELA−9 genes, ELK1–8 genes, FOXO3–7 genes, and, finally, HOXB4 and MEF2A−5 genes for each (data not shown).

We draw two principal conclusions from the results of “ChEA 2016” analysis. The first conclusion is that, in fact, all the genes, potentially implicated in maintaining stem-like phenotype of Krebs-2 TAMRA+ cells, can be activated under generalized cellular stress conditions. And the second one is that such an activation can be mediated both by induction of stemness by SOX2, OCT4/POU5F1, Nanog, KLF4, and c-Myc factors, and by direct action of specific mediators of cellular response to hypoxia-xenobiotics-oxidative stress. Yet the presence of binding sites for certain transcription factors does not necessarily ensure the transcriptional activation that depends significantly on general epigenetic/physiological context. It presumes the necessity of complex approach. As we already mentioned above, there are experimental evidences that a number of genes from our list are activated under stress conditions. The analysis of binding sites, respectively, suggests the possible mechanisms of such activation and allows us to extrapolate these mechanisms to other “stem genes.”

### If there is a third way: discussion

The issue of the mechanisms of CSCs origination as well as of means they use to self-maintain and increase their population in developing malignant neoplasms is one of the most important for modern oncology, as it is key for the development of methods of antitumor therapy.

The classical model for the formation of CSCs subpopulation is based on the ability of pluripotent cells to divide symmetrically, as the main way of self-renewal of the population (Franco et al., 2016; Rich, 2016). Moreover, CSCs possess the additional ability to retain their “pluripotent” properties outside of the “stem niche” conditions as well as the ability for amoeboid migration characteristic for most of poorly differentiated cells (Sakamoto et al., 2011). It ensures a uniform distribution of the initiating cells newly formed after symmetrical division throughout the tumor volume and, respectively, provides conditions for the continuous exponential growth of the tumor mass.

The model of stemness induction under conditions of generalized cellular stress we have proposed, complements the classical model and allows to resolve certain discrepancies in the available experimental data with the model “symmetrical division-migration.” At this, it should be emphasized that our concept of generalized cellular stress is not limited to the factors listed above and can be extended with such components as inflammation, ionizing radiation, heat shock, etc. Moreover, this model can also possibly explain the carcinogenic effect of chronic oxidative stress, inflammation and the action of carcinogenic xenobiotics through *de novo* induction of “pluripotency” followed by transformation into malignancy.

Simultaneous existence of two independent and complementary mechanisms for the formation and maintenance of CSCs subpopulation implies that there may be a third and a fourth variant(s). To complete the picture of possible mechanisms of CSCs origination, other hypotheses also need to be mentioned.

One of the hypotheses explains the phenomenon of CSCs *de novo* emergence due to genetic instability that is inherent characteristic of tumor cells. Formation of cells with a stem-like phenotype evenly dispersed throughout the volume of the tumor mass is believed to be the one of possible consequences of this instability (Lagasse, 2008). However, this explanation has a significant drawback, as it is barely consistent with the fact that tumors retain their histological and biochemical properties, and, accordingly, the overall transcriptional profile during development, as well as upon metastasis and transplantation into model animals (Franzén et al., 1997; Süsskind et al., 2017). This fact testifies to the persistence of a certain “genetic individuality” of cells that drive tumor growth, which to significant extent contradicts the stochastic model of the formation of a tumorigenic population due to genetic instability.

Another possible mechanism for the formation of a pluripotent phenotype in tumor cells could be the phenomenon of “genometastasis” (García-Casas et al., 2017). It is supposed that extracellular double-stranded DNA released from cells that have undergone apoptosis or necrosis, both primary and secondary, can be internalized by cancer cells that have passed the first stages of commitment/differentiation, but still retained such a basic feature of CSC as the ability to capture fragments of extracellular double-stranded DNA. The occurrence of DNA with certain genetic or structural features in internal compartments of such cells can lead to a restoration of the pluripotent potential of the committed cells and their reversible conversion into new CSCs. The proposed “reversive mechanism” does not contradict the proposed concept of the stemness induction under the generalized cellular stress, but, somehow, complements it, since the action of factors of generalized cellular stress is always accompanied by intensive death of cancer cells, which results in an excessive amount of extracellular double-stranded DNA (Wen et al., 2017). This hypothesis addresses the mechanism for retransformation of the early committed progeny of existing CSCs. The main disadvantage of this model, as well as of the previous one, based on genetic instability, is indeterminacy and randomness of the results of events occurring during the “genometastasis” (multiple mutations, random genetic composition of the internalized DNA etc).

Another intriguing model of CSC formation is the “Blebbishield emergency program.” It was found that cancer cells undergoing apoptosis can avoid cell death by evoking this program. During this process, one of the apoptotic bodies becomes a center of aggregation for other ones that results in the formation of so-called “*Blebbishield*” that, in turn, further transforms into a new CSC. Such a newly formed CSC demonstrates a more aggressive tumorigenic behavior and can even fuse with immunity cells. As a result of all these transformations, the new secondary tumor with significantly more aggressive characteristics arises (Jinesh and Kamat, 2016, 2017).

In general, all the hypotheses considered, starting with genetic instability and ending with the fusion of apoptotic bodies, describe the formation of pluripotent/stem phenotype of tumor cells as a probabilistic event with unpredictable results, somehow or other related to changes in their genetic material. The fluctuations in the percentage of CSCs we have observed in experimental tumors (Potter et al., 2016a) suggests that the main cause of “dynamic stemness” is not genomic but epigenetic changes.

The model we proposed for stemness induction in response to the components of generalized cellular stress, namely hypoxia, oxidative stress and the action of xenobiotics, apparently describes some basic mechanisms of the cellular response to stress. It can be presumed that CSCs serve as a kind of “Emergency service” for tumors, emerging *de novo* and ensuring their survival under unfavorable conditions. With all this, a number of questions remain, and the main one is why the proportion of CSCs relative to the entire mass of the tumor remains rather low despite the stress conditions? Moreover, it is not clear how long CSCs can sustain a stem-like phenotype, and whether stemness maintenance depends on external conditions or gradually fades regardless of the presence/absence of inducing agents?

Assuming all of the above, we have to admit that the significant majority of existing anti-tumor pharmaceutical and radiotherapy schemes lead to the formation of generalized cellular stress conditions, and, therefore, are likely to induce *de novo* formation of CSCs in the total mass of nonstem tumor cells (Chang, 2016). Perhaps this explains the fact that despite a certain progress, the overall effectiveness of cancer treatment remains extremely unsatisfactory, and cancer remains one of the leading causes of mortality in the world.

## Author contributions

YE performed the analysis, interpreted the data, and drafted the manuscript. AP interpreted the data and drafted the manuscript. EP and ED interpreted the data. OE performed the analysis. OT, AO, and EC participated in the study design. NK coordinated all work. SB conceived the study, participated in its design, coordinated and drafted the manuscript. All authors read and approved the final manuscript.

### Conflict of interest statement

The authors declare that the research was conducted in the absence of any commercial or financial relationships that could be construed as a potential conflict of interest.

## Abbreviations

CSC: cancer stem cell
TAMRA: carboxytetramethylrhodamine, fluorescent dye.
